# COVID-19–Related Internet Search Patterns Among People in the United States: Exploratory Analysis

**DOI:** 10.2196/22407

**Published:** 2020-11-23

**Authors:** Tony S Shen, Aaron Z Chen, Patawut Bovonratwet, Carol L Shen, Edwin P Su

**Affiliations:** 1 Hospital for Special Surgery New York, NY United States; 2 Weill Cornell Medical College New York, NY United States; 3 NewYork-Presbyterian Morgan Stanley Children’s Hospital New York, NY United States

**Keywords:** COVID-19, pandemic, internet, infodemic, infodemiology, infoveillance, natural language processing, NLP, health information, information seeking

## Abstract

**Background:**

The internet is a well-known source of information that patients use to better inform their opinions and to guide their conversations with physicians during clinic visits. The novelty of the recent COVID-19 outbreak has led patients to turn more frequently to the internet to gather more information and to alleviate their concerns about the virus.

**Objective:**

The aims of the study were to (1) determine the most commonly searched phrases related to COVID-19 in the United States and (2) identify the sources of information for these web searches.

**Methods:**

Search terms related to COVID-19 were entered into Google. Questions and websites from Google web search were extracted to a database using customized software. Each question was categorized into one of 6 topics: clinical signs and symptoms, treatment, transmission, cleaning methods, activity modification, and policy. Additionally, the websites were categorized according to source: World Health Organization (WHO), Centers for Disease Control and Prevention (CDC), non-CDC government, academic, news, and other media.

**Results:**

In total, 200 questions and websites were extracted. The most common question topic was transmission (n=63, 31.5%), followed by clinical signs and symptoms (n=54, 27.0%) and activity modification (n=31, 15.5%). Notably, the clinical signs and symptoms category captured questions about myths associated with the disease, such as whether consuming alcohol stops the coronavirus. The most common websites provided were maintained by the CDC, the WHO, and academic medical organizations. Collectively, these three sources accounted for 84.0% (n=168) of the websites in our sample.

**Conclusions:**

In the United States, the most commonly searched topics related to COVID-19 were transmission, clinical signs and symptoms, and activity modification. Reassuringly, a sizable majority of internet sources provided were from major health organizations or from academic medical institutions.

## Introduction

Since its emergence in late 2019 in Wuhan, China, COVID-19, the disease caused by the novel coronavirus SARS-CoV-2 has drastically impacted daily life around the world [1,2]. Among the changes to the public sphere include government-imposed lockdowns of businesses, schools, and universities, designed to mitigate the spread of the disease and to alleviate the significant strain on health care systems [2]. As many continue to stay at home due to the COVID-19 pandemic, internet use has become an increasingly dominant part of daily life. In a recent poll, a majority of Americans considered the internet “essential” during this time [3]. Nearly all major internet services have seen increased traffic since early March 2020 [4]. Given the unprecedented nature of the pandemic, there is naturally much public uncertainty regarding COVID-19, and thus, many are turning to the internet to ask their questions and obtain information about the coronavirus.

Previous studies have shown that patients frequently use the internet to research their conditions and inform their discussions in clinic [5,6]. As physicians, insight into what topics patients are curious or anxious about may help guide and structure our interactions, leading to improved patient rapport and satisfaction. Additionally, with well-publicized recent examples of misinformation originating from many sources, including places of authority, it is paramount for physicians to collectively take responsibility to provide reliable and trustworthy information based on the best available evidence [7,8]. Thus, the aims of the present study were to (1) determine the most commonly searched phrases related to COVID-19 in the United States and (2) identify the sources of information for these web searches. In doing so, we believe that we can distill the collective curiosity of the internet-using public into useful information for physicians in clinic.

## Methods

Search terms related to COVID-19 were entered into Google web search using a clean-installed Google Chrome browser on May 30, 2020, in New York, NY. Google web search is by far the most widely used internet search engine in the United States [9]. In 2018, Google introduced a natural language processing algorithm, which greatly improved the ability of the search engine to identify clusters of search queries related to any given topic [10]. Due to this new technology, Google redirects all searches related to COVID-19, such as “COVID-19,” “coronavirus,” “coronavirus disease,” etc, to a centralized COVID-19 homepage. This search results page incorporates the location of the user’s search and generates a list of questions and websites that are frequently associated with the initial query. On each results page, the 200 most commonly asked questions were generated. The questions were downloaded to a database using a freely available program (Scraper, version 1.7). The specific question and web address were noted on the webpage by their unique XML Path Language (XPath) strings.

The questions were first categorized according to Rothwell’s classification system by a single trained reviewer [11,12]. This classification system was expanded into one of 6 topics—clinical signs and symptoms, treatment, transmission, cleaning methods, activity modification, and policy—which were chosen based on previously published studies that examined the web and social media concerns of users during the COVID-19 pandemic [13-15] (Table 1).

Activity modification consisted of questions regarding the effectiveness of various activities or lifestyle changes in preventing COVID-19. Policy included questions detailing local or national policy changes enacted in response to COVID-19, including questions about economic support. A full listing of the criteria for each topic category is listed in Table 1.

In line with previous studies, the websites were categorized according to source: World Health Organization (WHO), Centers for Disease Control and Prevention (CDC), non-CDC government, academic, news, and other media [16,17] (Table 1). Specifically, non-CDC government websites consisted of webpages directly maintained by a national governmental entity such as the National Institutes of Health (United States) or the National Health Service (United Kingdom). Academic websites were defined as an organization with a clear academic mission statement. Other media consisted of websites not described by one of the previous categories, including CNET, WebMD, and Wikipedia. A full listing of the criteria for each web source category is listed in Table 1.

**Table 1:** Question classification by topic and website categorization by source.

Variable			Description
**Question classification by topic**			
	Clinical signs and symptoms	Properties of SARS-CoV-2, signs and symptoms of COVID-19, prognosis, risk factors, severity, testing Example: can you have the coronavirus disease without a fever?	
	Treatment	Treatment strategies for COVID-19, including antiretrovirals and vaccines; also includes inquiries for unsupported treatments Example: Can antibiotics treat the coronavirus disease?	
	Transmission	Inquiries regarding specific routes of transmission for SARS-CoV-2 Example: Can coronavirus spread through mosquito bite?	
	Cleaning methods	Inquiries regarding specific methods of sanitation to limit spread of COVID-19 Example: Can ultraviolet light kill the coronavirus disease?	
	Activity modification	Questions regarding effectiveness of various activities or lifestyle changes in preventing COVID-19 Example: Can I walk my dog during quarantine?	
	Policy	Questions about local or national policy changes enacted in response to COVID-19; includes questions about economic support Example: Does everyone get a stimulus check?	
**Website categorization by source**			
	World Health Organization (WHO)	Website maintained by the WHO and hosted on the who.int domain Example: COVID-19 Situation Report (who.int)	
	Centers for Disease Control and Prevention (CDC)	Website maintained by the United States CDC and hosted on the cdc.gov domain Example: Travelers’ health (nc.cdc.gov/travel)	
	Government	Websites maintained by national, state, or local governmental organizations other than the WHO or CDC Example: New York State Governor’s Office (governor.ny.gov)	
	Academic	Websites maintained by an institution with a clear academic mandate, including universities, academic medical centers, and academic societies Example: ClevelandClinic.org, MayoClinic.org	
	News	Websites published by local, national, or international news media organizations Example: USAToday.com	
	Other media	Websites maintained by organizations not defined in the categories above Example: Wikipedia.org	

## Results

In total, 200 questions and their corresponding source of information were extracted; the top 25 questions are listed in Textbox 1. The most common question topic was transmission (n=63, 31.5%), followed by clinical signs and symptoms (n=54, 27.0%) and activity modification (n=31, 15.5%) (Table 2).

Most questions regarding the transmissibility of the coronavirus asked about specific modes of transmission such as spread through food, feces, air conditioning units, delivery packages, and mosquitoes. Interestingly, the clinical signs and symptoms category captured questions about myths associated with the disease, such as whether consuming alcohol stops the coronavirus. In the activity modifications category, there were many questions about staying at home, wearing masks, and managing pre-existing travel plans. The most commonly asked question—“Can antibiotics treat the coronavirus disease?”—was classified as treatment, which, in total, comprised 9% (n=18) of the searched questions.

**Top 25 questions associated with COVID-19.**

- Can antibiotics treat the coronavirus disease?
- Is headache a symptom of the coronavirus disease?
- Is there a vaccine for coronavirus?
- Are masks effective against the coronavirus disease?
- Can children pass on the coronavirus disease?
- Can coronavirus spread through mosquito bite?
- Can I stay at home if I have the coronavirus disease?
- Can the coronavirus disease spread faster in an air-conditioned house?
- Can the coronavirus disease spread through delivered packages?
- Can the coronavirus disease spread through feces?
- Can the coronavirus disease spread through food?
- Can you get coronavirus from talking to someone from a distance?
- Can you have the coronavirus disease without a fever?
- Does drinking alcohol kill the coronavirus?
- How long does the coronavirus stay on clothing?
- Is bleach an effective cleaning agent for the coronavirus disease?
- Is the coronavirus disease more severe than the flu?
- Should I accept packages from China?
- Should I cancel my trip due to coronavirus?
- Should I wear a face mask out in public?
- What antiviral drugs are available to treat the coronavirus disease?
- What is a pandemic?
- What is the recovery time for the coronavirus disease?
- Who gets a stimulus check?
- Are rashes a symptom of the coronavirus disease?
- Can hand sanitizer explode in a hot car?

**Table 2:** Frequencies and percentages associated with questions by topic and websites by source.

Variable		Frequency, n (%)	
**Questions by topic (n=200)**			
	Transmission		63 (31.5)
	Clinical signs and symptoms		54 (27.0)
	Activity modification		31 (15.5)
	Policy		22 (11.0)
	Treatment		18 (9.0)
	Cleaning methods		12 (6.0)
**Websites by source (n=200)**			
	Centers for Disease Control and Prevention		73 (36.5)
	Academic		48 (24.0)
	World Health Organization		47 (23.5)
	News		13 (6.5)
	Government		10 (5.0)
	Other media		9 (4.5)

With respect to sources of information, the most common websites provided were maintained by the CDC (n=73, 36.5%), academic medical organizations (n=48, 24.0%), and the WHO (n=47, 23.5%) (Table 2). With an additional 5% (n=10) of web information provided by a government source, an overwhelming majority of information (n=178, 89%) came from highly trustworthy web sources. However, the remaining 11% (n=22) of information came from either news or other media. In particular, 4.5% (n=9) of information came from web sources classified as other media, which included potentially erroneous sources of information such as Wikipedia.

## Discussion

### Principal Findings

In the midst of a highly unprecedented pandemic with significant economic and public health implications, the internet is a crucial source of information for the general public in order to guide their everyday life. As information is changing rapidly and is compounded by fallacies originating from places of authority, we believe that the pandemic highlights the role of physicians in providing patients the most reliable information based on the highest quality of evidence. Thus, the present study effectively characterized the intellectual curiosity of millions of Americans into 6 easily categorizable groups and demonstrated the origin of the general public’s sources of information.

Previous studies have examined search and Twitter trends related to the COVID-19 pandemic from regions around the world, including the United States, China, Italy, and Spain [13-15,18-20]. In April, Husnayain et al [14] examined Google search trends in Taiwan, effectively noting that searches for handwashing drastically increased after a perceived face mask shortage in the country. More recently, Rovetta et al [19] examined the Google search trends in Italy, and characterized the most common search terms in the country, including “face mask,” “disinfectant,” “symptoms of the coronavirus,” “health bulletin,” and “vaccines.” In the United States, Chen et al [18] examined over 100 million tweets to track social media conversations about the COVID-19 pandemic. However, to our knowledge, no study has examined internet search patterns related to COVID-19 in the United States. This question is of utmost importance for several reasons. First, the United States represents not only the highest COVID-19 burden in the world, but is also a country where recent well-publicized examples of misinformation originated from the head of state [8]. Second, while Twitter effectively captures a significant source of information, it is by no means comprehensive, and the platform appeals to a select audience [21].

Thus, the present study revealed that the most commonly searched criteria about COVID-19 included information about transmission, clinical signs and symptoms, and activity modification. Understanding what matters to our patients should compel us to be well informed on these topics. We believe that as physicians, we should collectively take responsibility to provide reliable information based on the best available evidence [22]. Even if we do not regularly manage patients with COVID-19, at the very least we should be prepared to answer the most common clinical questions asked online such as modes of transmission or the status of a vaccine. Although many answers may be obvious to us, there remain many questions that are active areas of study for which we must remain up to date. Regardless of the specific details of our individual practice, we should always have the willingness to learn and the preparation to answer these commonly asked questions.

Further, the present study revealed that the sizable majority of internet sources provided were from major health organizations or from academic medical institutions. While we find this to be encouraging, we must remember that our patients also consume information from multiple other sources. The social media “echo chamber” phenomenon is an active area of study for sociologists and computer scientists and has been shown to rapidly propagate rumors or misinformation on a mass scale [23-25]. Again, we believe that physicians should take responsibility for providing the best-quality information in the domains in which we hold influence. On a larger scale, perhaps there is a role for physicians to learn and adapt techniques employed by marketers and politicians to better communicate medical information with the public. However, for most of us, we believe that our role is simply to understand the concerns of our patients regarding COVID-19, to remain informed ourselves, and to be ready to answer their questions.

### Limitations

There are several limitations to the present study. First, the COVID-19 pandemic is rapidly changing, and the results of the present study captured web searches as of May 2020. Due to the changes in pandemic characteristics, such as the emergence of new hotspots, it is entirely plausible that the focus of web searches has changed as well. In addition, the present study only captures the web searches of users in New York during May 2020, as the Google COVID-19 database generates search results based on the date of search and user location. Thus, we are unable to analyze trends in either. However, other published studies have examined Google searches in other regions of the world, including China, Taiwan, and Italy [13-15,19]. Thus, the results of the present study should be used in conjunction with those around the world to provide a more comprehensive view of the search patterns of citizens across the world. In addition, the present study makes use of Google’s coronavirus homepage, which generates the most commonly asked questions based on the specific user’s location and date of search. Due to the limitations of this feature, we are unable to compare trends in location and trends in time, which should be a direction for future studies. Lastly, Google web search was the only search database examined, and the present study fails to capture information from alternative search engines. However, as previously noted, Google is by far the most highly utilized search engine in the United States [9].

### Conclusion

People use Google Web Search to identify sources of information about COVID-19. In the United States, the most commonly searched topics related to COVID-19 were transmission, clinical signs and symptoms, and activity modification. Reassuringly, the majority of information in the present study came from highly reputable sources, including the CDC, academic websites, and the WHO.

## Abbreviations

CDC: Centers for Disease Control and Prevention
WHO: World Health Organization
XPath: XML Path Language
